# Supplementary material for: Mechano-calcium and mechano-electric feedbacks in the human cardiomyocyte analyzed in a mathematical model
Source: J Physiol Sci. 2020 Feb 18;70:12. doi: 10.1186/s12576-020-00741-6 (PMC7028825; doi:10.1186/s12576-020-00741-6)
Supplement: Supplementary file 1 — Additional file 1. Details of the developed electromechanical TP + M model (equations, parameters, initial values of state variables). [file 12576_2020_741_MOESM1_ESM.pdf]

'ten Tusscher – Panfilov' + Mechanics Model

State variables

	Definition	Initial value	Unit
V	membrane potential	-84.79	mV
d	voltage-dependent activation gate (i_{CaL})	$3.575 \cdot 10^{-5}$	dimensionless
$f2$	fast voltage-dependent inactivation gate (i_{CaL})	0.9994	dimensionless
$fCass$	intracellular Ca^{2+} inactivation gate (i_{CaL})	0.99998	dimensionless
f	slow voltage-dependent inactivation gate (i_{CaL})	0.9843	dimensionless
R_{prime}	proportion of closed I_{rel} channels	0.9903	dimensionless
Ca_{SR}	sarcoplasmic reticulum Ca^{2+} concentration	3.26	mM
Ca_{ss}	subspace Ca^{2+} concentration	0.00018	mM
$CaTnC$	Ca^{2+}-troponin complexes concentration	0.00112	mM
h	fast inactivation gate (i_{Na})	0.7326	dimensionless
j	slow inactivation gate (i_{Na})	0.7314	dimensionless
m	activation gate (i_{Na})	0.00188	dimensionless
$Xr1$	activation gate (i_{Kr})	0.00023	dimensionless
$Xr2$	inactivation gate (i_{Kr})	0.46662	dimensionless
Xs	activation gate (i_{Ks})	0.00336	dimensionless
r	voltage-dependent activation gate (i_{to})	$2.6 \cdot 10^{-8}$	dimensionless
s	voltage-dependent inactivation gate (i_{to})	0.999998	dimensionless
K_i	intracellular K^+ concentration	134.42	mM
Na_i	intracellular Na^+ concentration	11.16	mM
Ca_i	intracellular Ca^{2+} concentration	0.000076	mM
l_1	deformation of CE against its slack length	0.386	μm
l_2	deformation of PE against its slack length	0.386	μm
l_3	deformation of XSE against its slack length	0.058	μm
v	velocity of CE deformation	0	μm/ms
w	velocity of PE deformation	0	μm/ms
N	cross-bridges concentration	$5.69 \cdot 10^{-6}$	dimensionless

Constants

	Definition	Value	Unit
$stim_{amp}$	amplitude of i_{stim}	52	pA/pF
$stim_{dur}$	duration of i_{stim}	1	ms
$stim_{per}$	periodicity of i_{stim}	1000	ms
$stim_{start}$	start of i_{stim}	10	ms
F	Faraday constant	96485.3415	C/M
R	gas constant	8314.472	mJ·K^{-1}·M^{-1}
T	temperature	310	K
Cm	cell capacitance	0.185	μF
V_c	cytoplasmic volume	0.016404	mm^3

Constants (continued)

	Definition	Value	Unit
V_{sr}	sarcoplasmic reticulum volume	0.001094	mm^3
V_{ss}	subspace volume	0.0000547	mm^3
Ca_o	intracellular Ca^{2+} concentration	2	mM
K_o	extracellular K^+ concentration	5.4	mM
Na_o	extracellular Na^+ concentration	140	mM
g_{CaL}	maximal i_{CaL} conductance	0.00005	l/(F· s)
g_{bCa}	maximal i_{bCa} conductance	0.000592	nS/pF
K_{pCa}	Ca_i half-saturation constant of i_{pCa}	0.0005	mM
g_{pCa}	maximal i_{pCa} conductance	0.1238	pA/pF
Buf_{sr}	total sarcoplasmic buffer concentration	10	mM
Buf_{ss}	total subspace buffer concentration	0.4	mM
Buf_c	total (except $CaTnC$) cytoplasmic buffer concentration	0.13	mM
EC	Ca_{SR} half-saturation constant of k_{casr}	1.5	mM
$K_{buf_{sr}}$	Ca_{SR} half-saturation constant for sarcoplasmic buffer	0.3	mM
$K_{buf_{ss}}$	Ca_{SS} half-saturation constant for subspace buffer	0.00025	mM
K_{buf_c}	Ca_i half-saturation constant for cytoplasmic buffer	0.00085	mM
V_{leak}	maximal I_{leak} conductance	0.00036	ms^{-1}
V_{rel}	maximal I_{rel} conductance	0.1224	ms^{-1}
V_{xfer}	maximal I_{xfer} conductance	0.00456	ms^{-1}
K_{up}	half-saturation constant of I_{up}	0.00025	mM
V_{maxup}	maximal I_{up} conductance	0.00765	mM/ms
$k1_{prime}$	R to O and R1 to I I_{rel} transition rate	0.15	$mM^{-2}\cdot ms^{-1}$
$k2_{prime}$	O to I and R to R1 I_{rel} transition rate	0.045	$mM^{-1}\cdot ms^{-1}$
$k3$	O to I and R to R1 I_{rel} transition rate	0.06	ms^{-1}
$k4$	I to O and R1 to I I_{rel} transition rate	0.005	ms^{-1}
max_{sr}	maximum value of k_{casr}	2.5	dimensionless
min_{sr}	minimum value of k_{casr}	1	dimensionless
Π_{min}	parameter of Π_{N_A} function	0.02	dimensionless
s_c	parameter of $N_A(CaTnC, N)$ function	1.0	dimensionless
TnC_{tot}	total concentration of TnC	0.07	mM
k_A	cooperativity parameter	28.0	mM^{-1}
a_{off}	maximum rate constant for $CaTnC$ dissociation	0.17	ms^{-1}
a_{on}	rate constant for $CaTnC$ association	35.0	$mM^{-1}\cdot ms^{-1}$
g_{Na}	maximal i_{Na} conductance	14.838	nS/pF
g_{bna}	maximal i_{bNa} conductance	0.00029	nS/pF
g_{K1}	maximal i_{K1} conductance	5.405	nS/pF
g_{pK}	maximal i_{pK} conductance	0.0146	nS/pF
g_{Kr}	maximal i_{Kr} conductance	0.153	nS/pF

Constants (continued)

	Definition	Value	Unit
P_{kna}	relative i_{Ks} permeability to Na^+	0.03	dimensionless
g_{Ks}	maximal i_{Ks} conductance	0.392	nS/pF
g_{to}	maximal i_{to} conductance	0.294	nS/pF
K_{NaCa}	maximal i_{NaCa}	10000	pA/pF
K_{sat}	saturation factor for i_{NaCa}	0.1	dimensionless
Km_{Ca}	Ca_i half-saturation constant for i_{NaCa}	1.38	mM
Km_{Nai}	Na_i half-saturation constant for i_{NaCa}	87.5	mM
α	factor enhancing outward nature of i_{NaCa}	1	dimensionless
γ	voltage dependence parameter of i_{NaCa}	0.35	dimensionless
K_{mNa}	Na_i half-saturation constant for i_{NaK}	40	mM
K_{mK}	K_o half-saturation constant for i_{NaK}	1	mM
P_{NaK}	maximal i_{NaK}	2.724	pA/pF
λ	scale parameter of F_{CE}	350.0	AFU
α_1	exponential coefficient of F_{SE}	14.6	μm^{-1}
β_1	linear coefficient of F_{SE}	4.2	AFU
α_2	exponential coefficient of F_{PE}	14.6	μm^{-1}
β_2	linear coefficient of F_{PE}	0.009	AFU
α_3	exponential coefficient of F_{XSE}	55.0	μm^{-1}
β_3	linear coefficient of F_{XSE}	0.11	AFU
α_{vp_l}	exponential coefficient of F_{VS_1}	16.0	μm^{-1}
α_{vp_s}	exponential coefficient of F_{VS_1}	16.0	μm^{-1}
β_{vp_l}	linear coefficient of F_{VS_1}	0.1	AFU$\cdot$ ms/μm
β_{vp_s}	linear coefficient of F_{VS_1}	0.1	AFU$\cdot$ ms/μm
α_{vs_l}	exponential coefficient of F_{VS_2}	46.0	μm^{-1}
α_{vs_s}	exponential coefficient of F_{VS_2}	39.0	μm^{-1}
β_{vs_l}	linear coefficient of F_{VS_2}	20.0	AFU$\cdot$ ms/μm
β_{vs_s}	linear coefficient of F_{VS_2}	60.0	AFU$\cdot$ ms/μm
v_{max}	parameter of p function	0.0055	μm/ms
a	parameter of p function	0.25	dimensionless
d_h	parameter of P_{star} function	0.5	dimensionless
α_P	parameter of G_{star} function	4.0	dimensionless
α_G	parameter of G_{star} function	1.0	dimensionless
k_μ	parameter of M_A function	0.6	dimensionless
μ	parameter of M_A function	3.3	dimensionless
g_1	parameter of n_1 function	0.6	μm^{-1}
g_2	parameter of n_1 function	0.52	dimensionless
$n1_A$	parameter of n_1 function	0.5	dimensionless
$n1_B$	parameter of n_1 function	55	μm
$n1_C$	parameter of n_1 function	1	dimensionless
$n1_Q$	parameter of n_1 function	0.835	dimensionless
$n1_K$	parameter of n_1 function	1	dimensionless
$n1_\nu$	parameter of n_1 function	5	dimensionless
S_0	parameter of L_{oz} function	1.14	μm
S_{055}	parameter of L_{oz} function	0.55	μm
S_{046}	parameter of L_{oz} function	0.46	μm

Constants (continued)

	Definition	Value	Unit
κ_0	parameter of κ function	2.1	dimensionless
κ_1	parameter of κ function	0.55	dimensionless
κ_2	parameter of κ function	0.0	dimensionless
m_0	fraction of strongly attached Xb in steady state isometric conditions	0.9	dimensionless
q_1	parameter of q function	0.0173	ms^{-1}
q_2	parameter of q function	0.259	ms^{-1}
q_3	parameter of q function	0.0173	ms^{-1}
q_4	parameter of q function	0.015	ms^{-1}
α_Q	parameter of q function	10.0	dimensionless
β_Q	parameter of q function	5.0	dimensionless
x_{st}	parameter of q function	0.964285	dimensionless
$r0$	preload	2.552	AFU (for $L_{init} = 90\% L_{max}$)
F_{aft}	afterload	6.89	AFU (for $10\% F_{isom}$)
k_{phys_rel}	parameter of V_{phys_rel} function	0.05	ms^{-1}
a_{phys_rel}	parameter of V_{phys_rel} function	*calculated*	μm
t_{phys_rel}	parameter of V_{phys_rel} function	*calculated*	ms
per_{phys_rel}	parameter of V_{phys_rel} function	230	ms

i_{stim}, stimulating current.

Calcium currents:
i_{CaL}, L-type Ca^{2+} current;
i_{bCa}, background Ca^{2+} current.

Calcium translocations:
I_{rel}, Ca^{2+} release from the sarcoplasmic reticulum (SR) via ryanodine receptors to the subspace (SS);
I_{xfer}, Ca^{2+} diffusion from SS to the cytoplasm (C);
I_{leak}, a small Ca^{2+} leakage from the SR to the cytoplasm;
I_{up}, Ca^{2+} pumping from the cytoplasm to the SR.
O, open conducting state of I_{rel}; R, resting closed state of I_{rel}; I, inactivated closed state of I_{rel}; R1, resting inactivated closed state of I_{rel}.

Calcium buffers:
CaB, buffering by other than $CaTnC$ intracellular ligands;
$CaTnC$, Ca^{2+}-troponin C complexes complexes;
Π_{N_A}, dependence defining cooperativity of the contractile proteins;
N_A, average fraction of the attached cross-bridges per one $CaTnC$ complex;
$CaSRB$, calcium buffering in SR;
$CaSSB$, subspace calcium buffering.

Sodium currents:
i_{Na}, fast Na^+ current;
i_{bNa}, background Na^+ current.

Potassium currents:
i_{K1}, inward rectifier K^+ current;
i_{to}, transient outward current;
i_{Kr}, i_{Ks}, rapid and slow delayed rectifier current;
i_{pK}, plateau K^+ current.

Pumps and exchangers:
i_{pCa}, sarcolemmal Ca^{2+} pump current;
i_{NaCa}, Na^+–Ca^{2+} exchanger current;
i_{NaK}, Na^+–K^+ pump current.

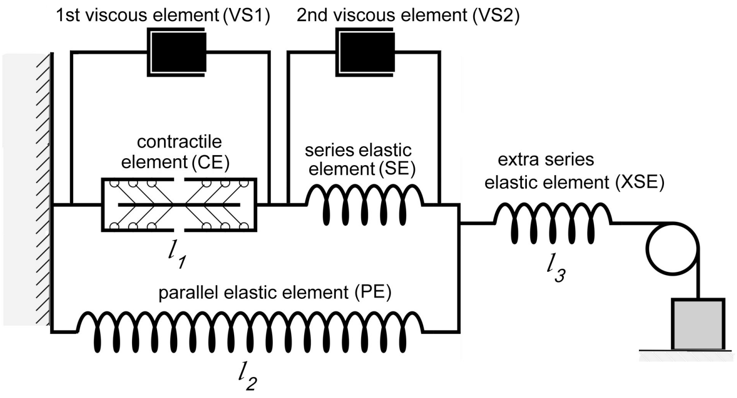

Figure 1: Rheological scheme in the TP+M model

Forces: AFU - arbitrary force unit;
F_{CE}, contractile element (sarcomere) (CE) force;
F_{SE}, serial elastic element (SE) force;
F_{PE}, parallel elastic element (PE) force;
F_{XSE}, extra serial elastic element (XSE) force;
F_{VS_1}, F_{VS_2}, viscous elements ($VS1$, $VS2$) forces.
l_1, deviation of contractile length from its slack length.
l_2, deviation of parallel elastic element length from its slack length.
l_3, deviation of extra series element length from its slack length.
$l = l_2 + l_3$, deviation of the sample length from its slack length.
p, dependence of the average cross-bridge force on the sarcomere shortening/lengthening velocity.
P_{star}, dependence of the steady-state sarcomere force on the sarcomere shortening/lengthening velocity.
G_{star}, dependence of the steady-state sarcomere stiffness on the velocity.
M_A, means end-to-end interaction between adjacent tropomyosin segments in the case if both of them affected by the respective $CaTnC$ complexes formation.
n_1, probability of that a myosin head can 'find' a vacant site on the actin filament.
L_{oz}, instantaneous length of thick and thin filament overlap zone.
κ, function required for variation the ratio between rates of cross-bridge attachment and detachment.

q, stationary relation 'stiffness-velocity' for the sample.
L_{init}, initial length of the sample.
L_{max}, corresponds to a sarcomere length equal to 2.23 μm.
F_{isom}, maximum of isometric force at given L_{init}.
V_{phys_rel}, dependence of sample length return during physiological relaxation.
k_{phys_rel}, physiological relaxation velocity.
a_{phys_rel}, physiological relaxation amplitude calculated as a difference between end-systolic and initial lengths of the sample.
t_{phys_rel}, time to start physiological relaxation fixed at the moment when $F_{sample} = r0$.
per_{phys_rel} period of physiological relaxation.

MODEL EQUATIONS

TNNP BLOCK (with modifications)

MEMBRANE POTENTIAL

$$i_{Stim} = \begin{cases} -stim_{amp} & \text{if } \left(time - \lfloor \frac{time}{stim_{per}} \rfloor \cdot stim_{per} \geq stim_{start}\right) \text{ and} \\ & \text{and } \left(time - \lfloor \frac{time}{stim_{per}} \rfloor \cdot stim_{per} \leq stim_{start} + stim_{dur}\right) \\ 0 & \text{otherwise} \end{cases}$$

$$\frac{\mathrm{d}V}{\mathrm{d}time} = \frac{-1}{1} \cdot (i_{K1} + i_{to} + i_{Kr} + i_{Ks} + i_{CaL} + i_{NaK} + i_{Na} + i_{b_{Na}} + i_{NaCa} + i_{b_{Ca}} + i_{p_K} + i_{p_{Ca}} + i_{Stim})$$

REVERSAL POTENTIALS

$$E_{Na} = \frac{R \cdot T}{F} \cdot \ln \frac{Na_o}{Na_i}$$

$$E_K = \frac{R \cdot T}{F} \cdot \ln \frac{K_o}{K_i}$$

$$E_{Ks} = \frac{R \cdot T}{F} \cdot \ln \frac{K_o + P_{kna} \cdot Na_o}{K_i + P_{kna} \cdot Na_i}$$

$$E_{Ca} = \frac{0.5 \cdot R \cdot T}{F} \cdot \ln \frac{Ca_o}{Ca_i}$$

L-type Ca^{2+} current

$$i_{CaL} = g_{CaL} \cdot d \cdot f \cdot f_2 \cdot f_{Cass} \cdot 4 \cdot \frac{(V-15) \cdot F^2}{R \cdot T} \cdot \frac{\left(0.25 \cdot Ca_{ss} \cdot e^{\frac{2\cdot(V-15)\cdot F}{R\cdot T}} - Ca_o\right)}{e^{\frac{2\cdot(V-15)\cdot F}{R\cdot T}} - 1}$$

L-type Ca^{2+} current. d gate

$$d_{inf} = \frac{1}{1 + e^{\frac{-8-V}{7.5}}}$$

$$\alpha_d = \frac{1.4}{1 + e^{\frac{-35-V}{13}}} + 0.25$$

$$\beta_d = \frac{1.4}{1 + e^{\frac{V+5}{5}}}$$

$$\gamma_d = \frac{1}{1 + e^{\frac{50-V}{20}}}$$

$$\tau_d = 1 \cdot \alpha_d \cdot \beta_d + \gamma_d$$

$$\frac{\mathrm{d}d}{\mathrm{d}time} = \frac{d_{inf} - d}{\tau_d}$$

L-type Ca^{2+} current. $f2$ gate

$$f2_{inf} = \frac{0.67}{1 + e^{\frac{V+35}{7}}} + 0.33$$

$$\tau_{f2} = 562 \cdot e^{\frac{-(V+27)^2}{240}} + \frac{31}{1 + e^{\frac{25-V}{10}}} + \frac{80}{1 + e^{\frac{V+30}{10}}}$$

$$\frac{\mathrm{d}f_2}{\mathrm{d}time} = \frac{f2_{inf} - f2}{\tau_{f2}}$$

L-type Ca^{2+} current. fCa_{SS} gate

$$fCass_{inf} = \frac{0.6}{1 + \left(\frac{Ca_{ss}}{0.05}\right)^2} + 0.4$$

$$\tau_{fCass} = \frac{80}{1 + \left(\frac{Ca_{ss}}{0.05}\right)^2} + 2$$

L-TYPE Ca^{2+} CURRENT. fCa_{SS} GATE (CONTINUED)

$$\frac{\mathrm{d}f_{Cass}}{\mathrm{d}time} = \frac{fCass_{inf} - fCass}{\tau_{fCass}}$$

L-TYPE Ca^{2+} CURRENT. f GATE

$$f_{inf} = \frac{1}{1 + e^{\frac{V+20}{7}}}$$

$$\tau_f = 1102.5 \cdot e^{\frac{-(V+27)^2}{225}} + \frac{200}{1 + e^{\frac{13-V}{10}}} + \frac{180}{1 + e^{\frac{V+30}{10}}} + 20$$

$$\frac{\mathrm{d}f}{\mathrm{d}time} = \frac{f_{inf} - f}{\tau_f}$$

Ca^{2+} BACKGROUND CURRENT

$$i_{b_{Ca}} = g_{bca} \cdot (V - E_{Ca})$$

Ca^{2+} PUMP CURRENT

$$i_{p_{Ca}} = \frac{g_{pCa} \cdot Ca_i}{Ca_i + K_{pCa}}$$

Ca^{2+} DYNAMICS

$$i_{rel} = V_{rel} \cdot O \cdot (Ca_{SR} - Ca_{ss})$$

$$i_{up} = \frac{Vmax_{up}}{1 + \frac{K_{up}^2}{Ca_i^2}}$$

$$i_{leak} = V_{leak} \cdot (Ca_{SR} - Ca_i)$$

$$i_{xfer} = V_{xfer} \cdot (Ca_{ss} - Ca_i)$$

$$O = \frac{k1 \cdot Ca_{ss}^2 \cdot R_{prime}}{k3 + k1 \cdot Ca_{ss}^2}$$

$$\frac{\mathrm{d}R_{prime}}{\mathrm{d}time} = -k2 \cdot Ca_{ss} \cdot R_{prime} + k4 \cdot (1 - R_{prime})$$

$$k1 = \frac{k1_{prime}}{kcasr}$$

$$k2 = k2_{prime} \cdot kcasr$$

$$kcasr = max_{sr} - \frac{max_{sr} - min_{sr}}{1 + \left(\frac{EC}{Ca_{SR}}\right)^2}$$

$$B_{Cabufc} = \frac{1}{1 + \frac{Buf_c \cdot K_{buf_c}}{(Ca_i + K_{buf_c})^2}}$$

$$B_{Cabufsr} = \frac{1}{1 + \frac{Buf_{sr} \cdot K_{buf_{sr}}}{(Ca_{SR} + K_{buf_{sr}})^2}}$$

$$B_{Cabufss} = \frac{1}{1 + \frac{Buf_{ss} \cdot K_{buf_{ss}}}{(Ca_{ss} + K_{buf_{ss}})^2}}$$

$$N_A = \frac{TnC_{tot} \cdot N \cdot sc}{L_{oz} \cdot CaTnC}$$

$$\Pi_{N_A} = \begin{cases} 1 & \text{if } N_A \leq 0 \\ \Pi_{min}^{N_A} & \text{if } N_A \leq 1 \\ \Pi_{min} & \text{otherwise} \end{cases}$$

$$\frac{\mathrm{d}CaTnC}{\mathrm{d}time} = a_{on} \cdot (TnC_{tot} - CaTnC) \cdot Ca_i - a_{off} \cdot e^{-k_A \cdot CaTnC} \cdot \Pi_{N_A} \cdot CaTnC$$

$$\frac{\mathrm{d}Ca_{sr}}{\mathrm{d}time} = B_{Cabufsr} \cdot (i_{up} - (i_{rel} + i_{leak}))$$

$$\frac{\mathrm{d}Ca_{ss}}{\mathrm{d}time} = B_{Cabufss} \cdot \left(\frac{-1 \cdot i_{CaL} \cdot Cm}{2 \cdot 1 \cdot V_{ss} \cdot F} + \frac{i_{rel} \cdot V_{sr}}{V_{ss}} - \frac{i_{xfer} \cdot V_c}{V_{ss}}\right)$$

$$CaB = \frac{Buf_c \cdot Ca_i}{Ca_i + K_{buf_c}}$$

$$CaSRB = \frac{Buf_{sr} \cdot Ca_{sr}}{Ca_{sr} + K_{buf_{sr}}}$$

$$CaSSB = \frac{Buf_{ss} \cdot Ca_{ss}}{Ca_{ss} + K_{buf_{ss}}}$$

FAST Na^+ CURRENT

$$i_{Na} = g_{Na} \cdot m^3 \cdot h \cdot j \cdot (V - E_{Na})$$

FAST Na^+ CURRENT. h GATE

$$h_{inf} = \frac{1}{\left(1 + e^{\frac{V+71.55}{7.43}}\right)^2}$$

$$\alpha_h = \begin{cases} 0.057 \cdot e^{\frac{-(V+80)}{6.8}} & \text{if } V < -40 \\ 0 & \text{otherwise} \end{cases}$$

$$\beta_h = \begin{cases} 2.7 \cdot e^{0.079 \cdot V} + 310000 \cdot e^{0.3485 \cdot V} & \text{if } V < -40 \\ \dfrac{0.77}{0.13 \cdot \left(1 + e^{\frac{V+10.66}{-11.1}}\right)} & \text{otherwise} \end{cases}$$

$$\tau_h = \frac{1}{\alpha_h + \beta_h}$$

$$\frac{\mathrm{d}h}{\mathrm{d}time} = \frac{h_{inf} - h}{\tau_h}$$

FAST Na^+ CURRENT. j GATE

$$j_{inf} = \frac{1}{\left(1 + e^{\frac{V+71.55}{7.43}}\right)^2}$$

$$\alpha_j = \begin{cases} \dfrac{\left(-25428 \cdot e^{0.2444 \cdot V} - 6.948 \cdot 10^{-6} \cdot e^{-0.04391 \cdot V}\right) \cdot (V + 37.78)}{1 + e^{0.311 \cdot (V+79.23)}} & \text{if } V < -40 \\ 0 & \text{otherwise} \end{cases}$$

$$\beta_j = \begin{cases} \dfrac{0.02424 \cdot e^{-0.01052 \cdot V}}{1 + e^{-0.1378 \cdot (V+40.14)}} & \text{if } V < -40 \\ \dfrac{0.6 \cdot e^{0.057 \cdot V}}{1 + e^{-0.1 \cdot (V+32)}} & \text{otherwise} \end{cases}$$

$$\tau_j = \frac{1}{\alpha_j + \beta_j}$$

$$\frac{\mathrm{d}j}{\mathrm{d}time} = \frac{j_{inf} - j}{\tau_j}$$

FAST Na^+ CURRENT. m GATE

$$m_{inf} = \frac{1}{\left(1 + e^{\frac{-56.86-V}{9.03}}\right)^2}$$

$$\alpha_m = \frac{1}{1 + e^{\frac{-60-V}{5}}}$$

$$\beta_m = \frac{0.1}{1 + e^{\frac{V+35}{5}}} + \frac{0.1}{1 + e^{\frac{V-50}{200}}}$$

$$\tau_m = 1 \cdot \alpha_m \cdot \beta_m$$

$$\frac{\mathrm{d}m}{\mathrm{d}time} = \frac{m_{inf} - m}{\tau_m}$$

Na^+ BACKGROUND CURRENT

$$i_{b_{Na}} = g_{bna} \cdot (V - E_{Na})$$

INWARD RECTIFIER K^+ CURRENT

$$\alpha_{K1} = \frac{0.1}{1 + e^{0.06 \cdot (V - E_K - 200)}}$$

$$\beta_{K1} = \frac{3 \cdot e^{0.0002 \cdot (V - E_K + 100)} + e^{0.1 \cdot (V - E_K - 10)}}{1 + e^{-0.5 \cdot (V - E_K)}}$$

$$xK1_{inf} = \frac{\alpha_{K1}}{\alpha_{K1} + \beta_{K1}}$$

$$i_{K1} = g_{K1} \cdot xK1_{inf} \cdot \sqrt{\frac{K_o}{5.4}} \cdot (V - E_K)$$

K^+ PLATEAU CURRENT

$$i_{p_K} = \frac{g_{pK} \cdot (V - E_K)}{1 + e^{\frac{25-V}{5.98}}}$$

RAPID TIME DEPENDENT K^+ CURRENT

$$i_{Kr} = g_{Kr} \cdot \sqrt{\frac{K_o}{5.4}} \cdot Xr1 \cdot Xr2 \cdot (V - E_K)$$

RAPID TIME DEPENDENT K^+ CURRENT. XR1 GATE

$$xr1_{inf} = \frac{1}{1 + e^{\frac{-26-V}{7}}}$$

$$\alpha_{xr1} = \frac{450}{1 + e^{\frac{-45-V}{10}}}$$

$$\beta_{xr1} = \frac{6}{1 + e^{\frac{V+30}{11.5}}}$$

$$\tau_{xr1} = 1 \cdot \alpha_{xr1} \cdot \beta_{xr1}$$

$$\frac{\mathrm{d}Xr1}{\mathrm{d}time} = \frac{xr1_{inf} - Xr1}{\tau_{xr1}}$$

RAPID TIME DEPENDENT K^+ CURRENT. XR2 GATE

$$xr2_{inf} = \frac{1}{1 + e^{\frac{V+88}{24}}}$$

$$\alpha_{xr2} = \frac{3}{1 + e^{\frac{-60-V}{20}}}$$

$$\beta_{xr2} = \frac{1.12}{1 + e^{\frac{V-60}{20}}}$$

$$\tau_{xr2} = 1 \cdot \alpha_{xr2} \cdot \beta_{xr2}$$

$$\frac{\mathrm{d}Xr2}{\mathrm{d}time} = \frac{xr2_{inf} - Xr2}{\tau_{xr2}}$$

SLOW TIME DEPENDENT K^+ CURRENT

$$i_{Ks} = g_{Ks} \cdot Xs^2 \cdot (V - E_{Ks})$$

SLOW TIME DEPENDENT K^+ CURRENT. XS GATE

$$xs_{inf} = \frac{1}{1 + e^{\frac{-5-V}{14}}}$$

$$\alpha_{xs} = \frac{1400}{\sqrt{1 + e^{\frac{5-V}{6}}}}$$

SLOW TIME DEPENDENT K^+ CURRENT. XS GATE (CONTINUED)

$$\beta_{xs} = \frac{1}{1 + e^{\frac{V-35}{15}}}$$

$$\tau_{xs} = 1 \cdot \alpha_{xs} \cdot \beta_{xs} + 80$$

$$\frac{\mathrm{d}Xs}{\mathrm{d}time} = \frac{xs_{inf} - Xs}{\tau_{xs}}$$

TRANSIENT OUTWARD K^+ CURRENT

$$i_{to} = g_{to} \cdot r \cdot s \cdot (V - E_K)$$

TRANSIENT OUTWARD K^+ CURRENT. R GATE

$$r_{inf} = \frac{1}{1 + e^{\frac{20-V}{6}}}$$

$$\tau_r = 9.5 \cdot e^{\frac{-(V+40)^2}{1800}} + 0.8$$

$$\frac{\mathrm{d}r}{\mathrm{d}time} = \frac{r_{inf} - r}{\tau_r}$$

TRANSIENT OUTWARD K^+ CURRENT. S GATE

$$s_{inf} = \frac{1}{1 + e^{\frac{V+20}{5}}}$$

$$\tau_s = 85 \cdot e^{\frac{-(V+45)^2}{320}} + \frac{5}{1 + e^{\frac{V-20}{5}}} + 3$$

$$\frac{\mathrm{d}s}{\mathrm{d}time} = \frac{s_{inf} - s}{\tau_s}$$

Na^+-Ca^{2+} EXCHANGER CURRENT

$$i_{NaCa} = \frac{K_{NaCa} \cdot \left(e^{\frac{\gamma \cdot V \cdot F}{R \cdot T}} \cdot Na_i^3 \cdot Ca_o - e^{\frac{(\gamma-1) \cdot V \cdot F}{R \cdot T}} \cdot Na_o^3 \cdot Ca_i \cdot \alpha \right)}{\left(Km_{Nai}^3 + Na_o^3\right) \cdot (Km_{Ca} + Ca_o) \cdot \left(1 + K_{sat} \cdot e^{\frac{(\gamma-1) \cdot V \cdot F}{R \cdot T}}\right)}$$

Na^{+}-K^{+} PUMP CURRENT

$$i_{NaK} = P_{NaK} \cdot \frac{K_o \cdot Na_i}{(K_o + K_{mk}) \cdot (Na_i + K_{mNa}) \cdot (1 + 0.1245 \cdot e^{\frac{-0.1 \cdot V \cdot F}{R \cdot T}} + 0.0353 \cdot e^{\frac{-V \cdot F}{R \cdot T}})}$$

Ca^{2+} DYNAMICS

$$\frac{\mathrm{d}Ca_i}{\mathrm{d}t} = B_{Cabufc} \cdot \left(\frac{(i_{leak} - i_{up}) \cdot V_{sr}}{V_c} + i_{xfer} - \frac{(i_{b_{Ca}} + i_{p_{Ca}} - 2 \cdot i_{NaCa}) \cdot Cm}{V_c \cdot F} - \frac{\mathrm{d}CaTnC}{\mathrm{d}t} \right)$$

K^{+} DYNAMICS

$$\frac{\mathrm{d}K_i}{\mathrm{d}time} = \frac{-1 \cdot (i_{K1} + i_{to} + i_{Kr} + i_{Ks} + i_{pK} + i_{Stim} - 2 \cdot i_{NaK})}{1 \cdot V_c \cdot F} \cdot Cm$$

Na^{+} DYNAMICS

$$\frac{\mathrm{d}Na_i}{\mathrm{d}time} = \frac{-1 \cdot (i_{Na} + i_{b_{Na}} + 3 \cdot i_{NaK} + 3 \cdot i_{NaCa})}{1 \cdot V_c \cdot F} \cdot Cm$$

MECHANICAL BLOCK

FORCE

$$F_{CE} = \lambda \cdot p_v \cdot N$$

$$F_{SE} = \beta_1 \cdot \left(e^{\alpha_1 \cdot (l_2 - l_1)} - 1\right)$$

$$F_{PE} = \beta_2 \cdot \left(e^{\alpha_2 \cdot l_2} - 1\right)$$

$$F_{XSE} = \beta_3 \cdot \left(e^{\alpha_3 \cdot l_3} - 1\right)$$

$$F_{VS_1} = k_{P_{vis}} \cdot v$$

$$F_{VS_2} = k_{S_{vis}} \cdot (w - v)$$

$$F_{sample} = F_{XSE}$$

CONTRACTION MODES

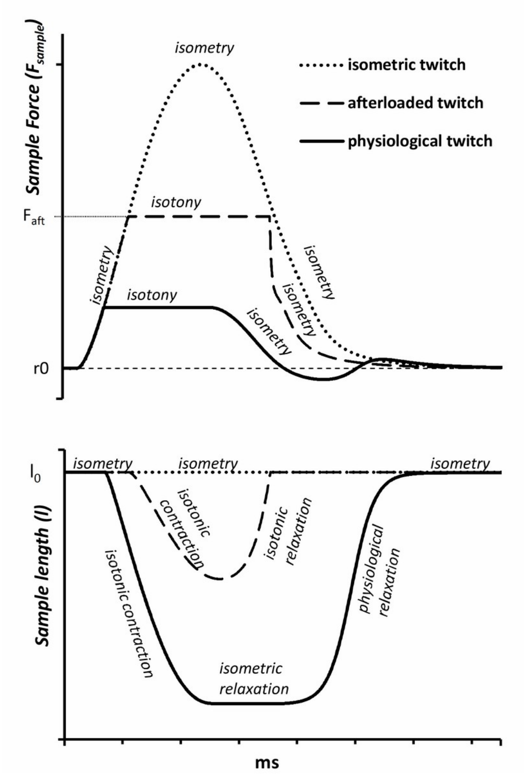

Figure 2: Contraction modes in the TP+M model

$$contraction_{mode} = \begin{cases} isometry & \\ isotony & \text{if } (F_{sample} > F_{aft}) \text{ and } \big(l \leq l_0 \cdot \big(1 + 1 \cdot 10^{-4}\big)\big) \\ isometric\_relaxation & \text{if } (l\ is\ end - systolic\ length) \text{ and } (F_{sample} > r0) \\ physiological\_relaxation & \text{starts after isometric relaxation when } (F_{sample} \leq r0) \end{cases}$$

LENGTH

$$l = l_2 + l_3$$

$$\frac{\mathrm{d}l_1}{\mathrm{d}time} = v$$

$$\frac{\mathrm{d}l_2}{\mathrm{d}time} = w$$

$$V_{phys\_rel} = \frac{a_{phys\_rel} \cdot k_{phys\_rel} \cdot e^{(-k_{phys\_rel} \cdot (t - (t_{phys\_rel} - per_{phys\_rel}/2)))}}{(1 + e^{(-k_{phys\_rel} \cdot (t - (t_{phys\_rel} - per_{phys\_rel}/2))})^2}$$

$$\frac{\mathrm{d}l_3}{\mathrm{d}time} = \begin{cases} -w & \text{if } contraction_{mode} = isometry\ and\ isometric\_relaxation \\ 0 & \text{if } contraction_{mode} = isotony \\ V_{phys\_rel} - w & \text{if } contraction_{mode} = physiological\_relaxation \end{cases}$$

$$alp_p = \begin{cases} \alpha_{vp_l} & \text{if } v \leq 0 \\ \alpha_{vp_s} & \text{otherwise} \end{cases}$$

$$k_{P_{vis}} = \begin{cases} \beta_{vp_l} \cdot e^{\alpha_{vp_l} \cdot l_1} & \text{if } v \leq 0 \\ \beta_{vp_s} \cdot e^{\alpha_{vp_s} \cdot l_1} & \text{otherwise} \end{cases}$$

$$\phi_\chi = \begin{cases} \frac{-\left(\lambda \cdot K_\kappa \cdot p_v + alp_p \cdot k_{P_{vis}} \cdot v^2 + \left(\alpha_2 \cdot \beta_2 \cdot e^{\alpha_2 \cdot l_2} + \alpha_3 \cdot \beta_3 \cdot e^{\alpha_3 \cdot l_3}\right) \cdot w\right)}{\lambda \cdot N \cdot p_{prime_v} + k_{P_{vis}}}, \\ \qquad \text{if } contraction_{mode} = isometry\ and\ isometric\_relaxation \\ \frac{-\left(\lambda \cdot K_\kappa \cdot p_v + alp_p \cdot k_{P_{vis}} \cdot v^2 + \alpha_2 \cdot \beta_2 \cdot e^{\alpha_2 \cdot l_2} \cdot w\right)}{\lambda \cdot N \cdot p_{prime_v} + k_{P_{vis}}}, \\ \qquad \text{if } contraction_{mode} = isotonic \\ \frac{-\left(\lambda \cdot K_\kappa \cdot p_v + alp_p \cdot k_{P_{vis}} \cdot v^2 + \alpha_2 \cdot \beta_2 \cdot e^{\alpha_2 \cdot l_2} \cdot w - \alpha_3 \cdot \beta_3 \cdot e^{\alpha_3 \cdot l_3} \cdot (V_{phys\_rel} - w)\right)}{\lambda \cdot N \cdot p_{prime_v} + k_{P_{vis}}}, \\ \qquad \text{if } contraction_{mode} = physiological\_relaxation \end{cases}$$

$$\frac{\mathrm{d}v}{\mathrm{d}time} = \phi_\chi$$

$$alp_s = \begin{cases} \alpha_{vs_l} & \text{if } w \leq v \\ \alpha_{vs_s} & \text{otherwise} \end{cases}$$

$$k_{S_{vis}} = \begin{cases} \beta_{vs_l} \cdot e^{\alpha_{vs_l} \cdot (l_2 - l_1)} & \text{if } w \leq v \\ \beta_{vs_s} \cdot e^{\alpha_{vs_s} \cdot (l_2 - l_1)} & \text{otherwise} \end{cases}$$

$$\frac{\mathrm{d}w}{\mathrm{d}time} = \begin{cases} \phi_\chi - alp_s \cdot (w - v)^2 - \dfrac{\alpha_1 \cdot \beta_1 \cdot e^{\alpha_1 \cdot (l_2 - l_1)} \cdot (w - v) + \left(\alpha_2 \cdot \beta_2 \cdot e^{\alpha_2 \cdot l_2} + \alpha_3 \cdot \beta_3 \cdot e^{\alpha_3 \cdot l_3}\right) \cdot w}{k_{S_{vis}}}, \\ \text{if } (contraction_{mode} = isometry \ and \ isometric\_relaxation) \\ \\ \dfrac{k_{S_{vis}} \cdot \left(\phi_\chi - alp_s \cdot (w - v)^2\right) - \alpha_1 \cdot \beta_1 \cdot e^{\alpha_1 \cdot (l_2 - l_1)} \cdot (w - v) - \alpha_2 \cdot \beta_2 \cdot e^{\alpha_2 \cdot l_2} \cdot w}{k_{S_{vis}}}, \\ \text{if } (contraction_{mode} = isotony) \\ \\ \phi_\chi - alp_s \cdot (w - v)^2 - \\ - \dfrac{\alpha_1 \cdot \beta_1 \cdot e^{\alpha_1 \cdot (l_2 - l_1)} \cdot (w - v) + \alpha_2 \cdot \beta_2 \cdot e^{\alpha_2 \cdot l_2} \cdot w - \alpha_3 \cdot \beta_3 \cdot e^{\alpha_3 \cdot l_3} \cdot (V_{phys\_rel} - w)}{k_{S_{vis}}}, \\ \text{if } (contraction_{mode} = physiological\_relaxation) \end{cases}$$

$$v_1 = \frac{v_{max}}{10}$$

$$\gamma_2 = \frac{a \cdot d_h \cdot \left(\frac{v_1}{v_{max}}\right)^2}{3 \cdot a \cdot d_h - \frac{(a+1) \cdot v_1}{v_{max}}}$$

$$P_{star} = \begin{cases} \frac{a \cdot \left(1 + \frac{v}{v_{max}}\right)}{a - \frac{v}{v_{max}}} & \text{if } v \leq 0 \\ 1 + d_h - \frac{d_h^2 \cdot a}{\frac{a \cdot d_h}{\gamma_2} \cdot \left(\frac{v}{v_{max}}\right)^2 + \frac{(a+1) \cdot v}{v_{max}} + a \cdot d_h} & \text{otherwise} \end{cases}$$

$$G_{star} = \begin{cases} 1 + \frac{0.6 \cdot v}{v_{max}} & \text{if } (v \leq 0) \\ \frac{P_{star}}{\frac{(0.4 \cdot a + 1) \cdot v}{a \cdot v_{max}} + 1} & \text{if } (0 < v) \text{ and } (v \leq v_1) \\ \frac{P_{star} \cdot e^{-\alpha_G \cdot \left(\frac{v - v_1}{v_{max}}\right)^{\alpha_P}}}{\frac{(0.4 \cdot a + 1) \cdot v}{a \cdot v_{max}} + 1} & \text{otherwise} \end{cases}$$

$$case_1 = \frac{a \cdot (0.4 + 0.4 \cdot a)}{v_{max} \cdot ((a+1) \cdot 0.4)^2}$$

$$case_2 = \frac{a \cdot 1 \cdot \left(1 + 0.4 \cdot a + \frac{1.2 \cdot v}{v_{max}} + 0.6 \cdot \left(\frac{v}{v_{max}}\right)^2\right)}{v_{max} \cdot \left(\left(a - \frac{v}{v_{max}}\right) \cdot \left(1 + \frac{0.6 \cdot v}{v_{max}}\right)\right)^2}$$

$$case_3 = \frac{0.4 \cdot a + 1}{a \cdot v_{max}}$$

$$case_4 = \frac{1}{v_{max}} \cdot e^{-\alpha_G \cdot \left(\frac{v - v_1}{v_{max}}\right)^{\alpha_P}} \cdot \left(\frac{0.4 \cdot a + 1}{a} + \alpha_G \cdot \alpha_P \cdot \left(1 + \frac{(0.4 \cdot a + 1) \cdot v}{a \cdot v_{max}}\right) \cdot \left(\frac{v - v_1}{v_{max}}\right)^{\alpha_P - 1}\right)$$

$$p_{prime_v} = \begin{cases} case_1 & \text{if } v \leq -v_{max} \\ case_2 & \text{if } (-v_{max} < v) \text{ and } (v \leq 0) \\ case_3 & \text{if } (0 < v) \text{ and } (v \leq v_1) \\ case_4 & \text{otherwise} \end{cases}$$

$$p_v = \frac{P_{star}}{G_{star}}$$

$$M_A = \frac{\left(\frac{CaTnC}{TnC_{tot}}\right)^{\mu} \cdot \left(1 + k_{\mu}^{\mu}\right)}{\left(\frac{CaTnC}{TnC_{tot}}\right)^{\mu} + k_{\mu}^{\mu}}$$

$$temp_{n1} = (g_1 \cdot l_1 + g_2) \cdot \left(n1_A + \frac{n1_K - n1_A}{\left(n1_C + n1_Q \cdot e^{-n1_B \cdot l_1}\right)^{\frac{1}{n1_{\nu}}}}\right)$$

$$n_1 = \begin{cases} 0 & \text{if } temp_{n1} < 0 \\ temp_{n1} & \text{if } temp_{n1} < 1 \\ 1 & \text{otherwise} \end{cases}$$

$$L_{oz} = \begin{cases} \frac{l_1 + S_0}{S_{046} + S_0} & \text{if } l_1 \leq S_{055} \\ \frac{S_0 + S_{055}}{S_{046} + S_0} & \text{otherwise} \end{cases}$$

$$\kappa = \begin{cases} \kappa_1 + \kappa_2 \cdot \frac{v}{v_{max}} & \text{if } v < 0 \\ \kappa_1 & \text{otherwise} \end{cases}$$

$$v_{st} = x_{st} \cdot v_{max}$$

$$q_v = \begin{cases} q_1 - \frac{q_2 \cdot v}{v_{max}} & \text{if } v \leq 0 \\ \frac{(q_4 - q_3) \cdot v}{v_{st}} + q_3 & \text{if } (v \leq v_{st}) \text{ and } (0 < v) \\ \frac{q_4}{\left(1 + \frac{\beta_Q \cdot (v - v_{st})}{v_{max}}\right)^{\alpha_Q}} & \text{otherwise} \end{cases}$$

$$k_{p_v} = \kappa \cdot \kappa_0 \cdot q_v \cdot m_0 \cdot G_{star}$$

$$k_{m_v} = \kappa_0 \cdot q_v \cdot (1 - \kappa \cdot m_0 \cdot G_{star})$$

$$K_{\kappa} = k_{p_v} \cdot M_A \cdot n_1 \cdot L_{oz} \cdot (1 - N) - k_{m_v} \cdot N$$

$$\frac{\mathrm{d}N}{\mathrm{d}time} = K_{\kappa}$$

Partial conversion from CellML 1.0 to TEX was done using COR (0.9.31.1409)
Copyright 2002-2018 Dr Alan Garny
